# Deubiquitination of CD36 by UCHL1 promotes foam cell formation

**DOI:** 10.1038/s41419-020-02888-x

**Published:** 2020-08-15

**Authors:** Xiaohong Xia, Qiong Xu, Mingke Liu, Xuke Chen, Xiaolin Liu, Jinchan He, Tumei Hu, Cuifu Yu, Hongbiao Huang, Shiming Liu, Ningning Liu

**Affiliations:** 1 Guangzhou Institute of Cardiovascular Disease, Guangdong Key Laboratory of Vascular Diseases, State Key Laboratory of Respiratory Disease, The Second Affiliated Hospital of Guangzhou Medical University, 510260 Guangzhou, China; 2 Guangzhou Municipal and Guangdong Provincial Key Laboratory of Protein Modification and Degradation, School of Basic Medical Sciences, Affiliated Cancer Hospital & Institute of Guangzhou Medical University, 511436 Guangzhou, China

**Keywords:** Atherosclerosis, Translational research

## Abstract

Atherosclerosis-associated cardiovascular diseases are main causes leading to high mortality worldwide. Macrophage-derived foam cell formation via uptaking modified lipoproteins is the initial and core step in the process of atherosclerosis. Meanwhile, scavenger receptor is indispensable for the formation of foam cells. UCHL1, a deubiquitinase, has been widely studied in multiple cancers. UCHL1 could be an oncogene or a tumor suppressor in dependent of tumor types. It remains unknown whether UCHL1 influences cellular oxLDL uptake. Herein we show that UCHL1 deletion significantly inhibits lipid accumulation and foam cell formation. Subsequently, we found that UCHL1 inhibitor or siRNA downregulates the expression of CD36 protein whereas SR-A, ABCA1, ABCG1, Lox-1, and SR-B1 have no significant change. Furthermore, the treatment of UCHL1 inhibition increases the abundance of K48-polyubiquitin on CD36 and the suppression of lipid uptake induced by UCHL1 deficiency is attenuated by blocking CD36 activation. Our study concluded that UCHL1 deletion decreases foam cell formation by promoting the degradation of CD36 protein, indicating UCHL1 may be a potential target for atherosclerosis treatment.

## Introduction

Cardiovascular diseases have been the most common cause of mortality and morbidity all over the world^1,2^. Atherosclerosis which is regarded as a chronic inflammatory disease is the fundamental pathological process underlying coronary artery disease (CAD) and stroke^3^. Atherosclerosis is caused mainly in large and medium arteries via recruiting immune cells to artery wall and disordering lipid metabolism^4,5^. The crucial early steps which recruited monocytes are differentiate into macrophages results from the retention of lipoproteins in subendothelial^6^. Subsequently, macrophages undergo modified lipoproteins (e.g., oxidized LDL, oxLDL) uptake regulated by scavenger receptors, including lectin-like oxLDL receptor 1 (Lox-1), CD36, or scavenger receptor-A (SR-A)^7–9^. When macrophages are as cholesterol-laden foam cells, inflammatory responses are triggered to accelerate plaque formation^9^. What is more, transformation of macrophage into foam cells is mediated by not only lipid uptake but also cholesterol efflux. Cholesterol efflux is a result of disturbance of scavenger receptor class B type 1 (SR-B) and ATP-binding cassette transporters A1 and G1 (ABCA1 and ABCG1)^10–12^. However, the mechanism regulated by the levels of these receptors remains incompletely understood.

Among these pivotal receptors, CD36 has a major role in the formation of foam cells^13,14^. It is reported that anti-CD36 antibody blocks oxLDL uptake by human monocyte-derived macrophages by 50%^15^. Moreover, monocytes which lack functional CD36 expression show remarkably downregulation of capacity for oxLDL binding^16^. Animal, which is genetic deletion of CD36 expression, exhibits decreased foam cell formation and induces the negative effect on the development of atherosclerosis in apo E^−/−^ mice^17^. On the other hand, there is a positive feedback loop between oxLDL and CD36 for foam cell formation, because of oxLDL-induced CD36 expression^18^. Therefore, altering CD36 expression is a possible project for foam cell formation. Interestingly, posttranslational modifications, such as ubiquitination, phosphorylation, were regulators in the regulation of protein stability^19^. Some reports have demonstrated that ubiquitin proteasome system (UPS) involved in the degradation of CD36^20^. Deubiquitinases (DUBs), a series of mediator in UPS, remove ubiquitin chains from their targeted proteins to stabilize their expressions. In our previously study, we have reported the deubiquitinase USP14 promotes CD36 stability via removing its ubiquition and inhibiting USP14 significantly decreases the formation of foam cell^21^.

UPS consists of 26S, which is comprises 19S and 20S. In the 19S regulatory particle exists three DUBs, including UCHL1, Rpn11, and USP14^22,23^. Ubiquitin C-terminal hydrolase 1 (UCHL1), a deubiquitinating enzymes, is one of the family of ubiquitin carboxy terminal hydrolase^24^. UCHL1 is overexpressed in some organs, such as gonads and diffuse neuroendocrine system. Alzheimer’s disease and Parkinson’s disease were associated with the mutation of UCHL1^25,26^. In addition, UCHL1 has a critical role in cancer treatment. UCHL1 is considered as an oncogene or a tumor inhibitor depending on which tumor^27–32^. However, the correlation between atherosclerosis and UCHL1 expression is not clear.

In view of these findings, we sought to confirm a possible function of UCHL1 in regulating foam cell formation in macrophages. In this study, we examined that inhibition or knockdown of UCHL1 suppressed lipid accumulation and foam cell formation via promoting degradation of CD36 cleaving K48-polyubiquitin. Hence, this finding provides a potent schedule for the therapy of cardiovascular disease.

## Results

### Inhibition of UCHL1 diminishes foam cell formation

**Fig. 1:**
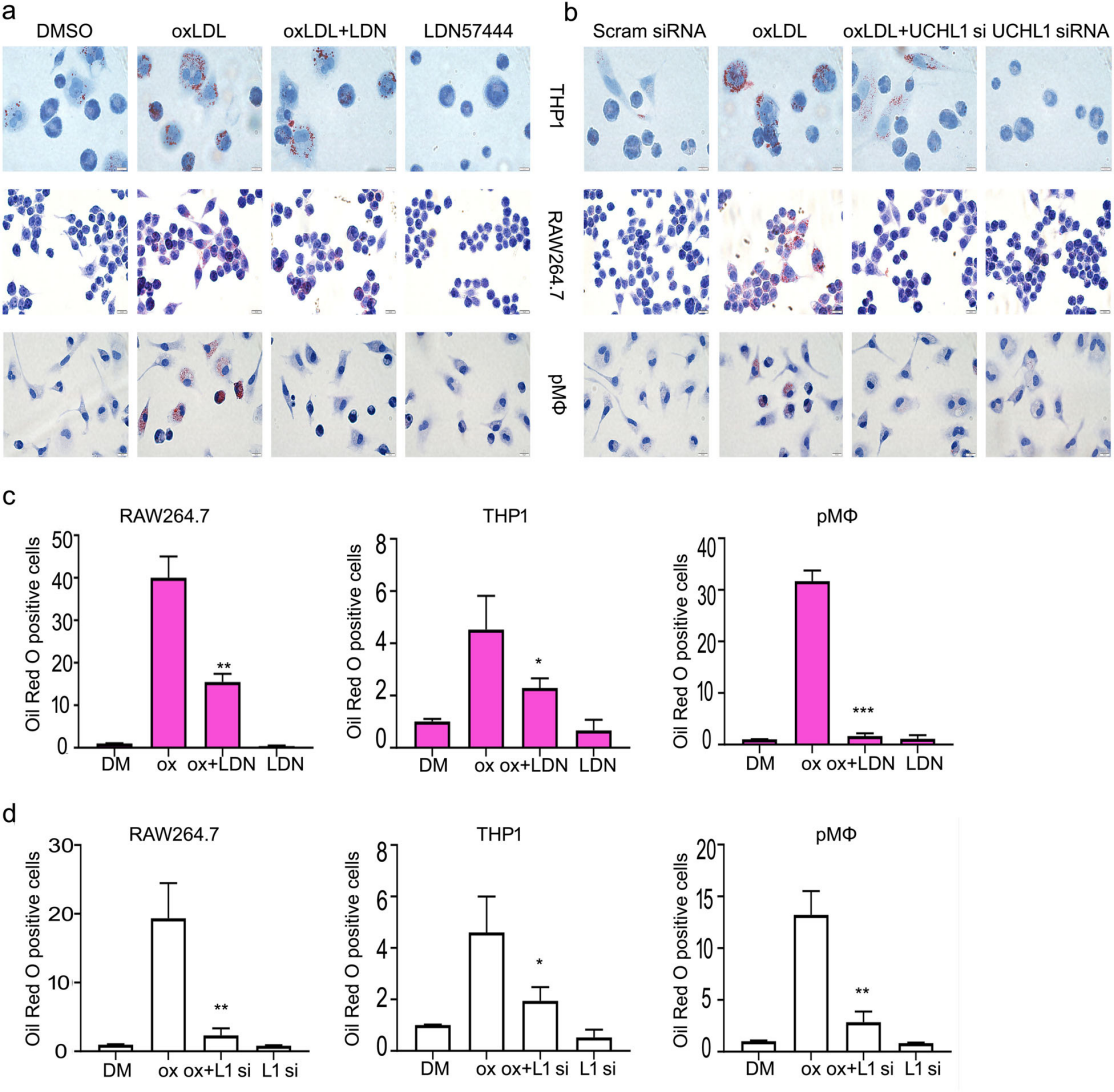
Inhibition of UCHL1 diminishes foam cell formation. **a** Macrophages were exposed to DMSO, oxLDL (50 μg/ml), LDN57444 (20 μM), and LDN57444 + oxLDL for 24 h. **b** UCHL1 siRNA retreated for 24 h, then adding oxLDL for additional 24 h in macrophages. Oil red O was applied to stain cells. The showed images were from three independent experiments. **c**, **d** The quantitative analysis of oil red O stained cells were counted. *p < 0.05, **p < 0.01, ***p < 0.001 versus oxLDL treatment group.

Firstly, to evaluate the effect of oxidized low-density lipoprotein (oxLDL) on macrophages, we applied 50 μg/ml oxLDL to incubated cells for 24 h in human THP1 macrophages, murine RAW264.7 macrophages and mouse primary peritoneal macrophages (pMΦ), which has been identified (Supplementary Fig. S1). And we found that intracellular lipids in oxLDL treatment group was more than that of in control using oil red O staining assays, suggesting that the macrophages were foamed and macrophage-derived foam cell formation (MFCF) model was successfully performed (Fig. 1a, b). Subsequently, we determined whether UCHL1 was involved in oxLDL-induced MFCF. As shown in Fig. 1a–d, inhibition of UCHL1 markedly attenuated the formation of foam cell. Interestingly, UCHL1 knockdown resulted in the similar results in the oil red O staining assay. The results showed that the contents of intracellular lipids were decreased by silencing UCHL1. These results indicated that the foam cell formation was suppressed by UCHL1 inhibitor or siRNA.

### UCHL1 deletion suppresses lipid uptake

**Fig. 2:**
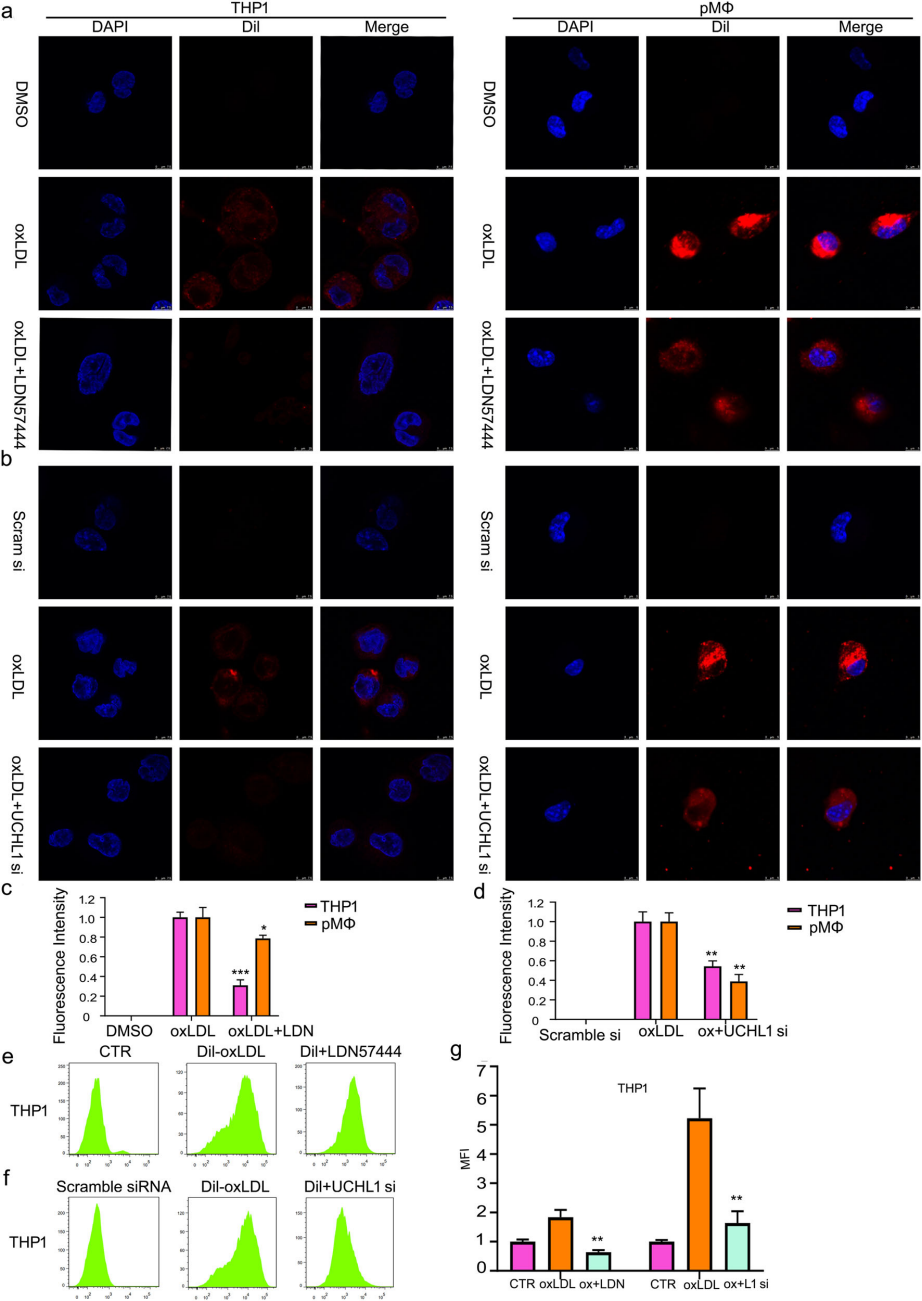
UCHL1 deletion suppresses lipid uptake. Macrophages were exposed to DMSO, oxLDL (50 μg/ml), LDN57444 (20 μM), and LDN57444 + oxLDL for 24 h. UCHL1 siRNA (50 nM) retreated for 24 h, then adding oxLDL (50 μg/ml) for additional 24 h in macrophages. Before cells were harvest for 6 h, Dil-oxLDL were used to incubate cells. The images were captured by confocal microscopy (**a**, **b**) and by flow cytometry (**e**, **f**). The showed images were from three experiments. **c**, **d**, **g** The quantitative analysis was presented. *p < 0.05, **p < 0.01, ***p < 0.001 versus oxLDL treatment group.

Since the formation of foam cells results from the oxLDL uptake, we further sought to exam UCHL1 function in regulating oxLDL entry into macrophages. To perform the analysis, we purchased oxLDL labeled by red fluorescent dye Dil. We found that co-culture with Dil-oxLDL for 6 h significantly increased the Dil-oxLDL uptake in THP1, RAW264.7 cells, and peritoneal macrophages (pMΦ) using confocal microscopy (Fig. 2a; Supplementary Fig. S2). Importantly, upon UCHL1 inhibition (LDN57444), the fluorescent intensity was decreased in Dil-oxLDL treated macrophages. Moreover, we found that oxLDL uptake was less than in UCHL1 silence + Dil-oxLDL treatments group than that of in Dil-oxLDL alone treatment (Fig. 2b). Indeed, we used ImageJ Pro to perform the quantitative analysis of fluorescent intensity. The results showed that UCHL1 regulated lipid uptake by macrophages (Fig. 2c, d). In addition to confocal microscopy, flow cytometry was used to detect the contribution of UCHL1 to oxLDL uptake. The results confirmed that UCHL1 inhibition or knockdown remarkably induced oxLDL uptake decrease in human THP1 macrophages (Fig. 2e–g). These results demonstrated that the enhanced oxLDL uptake by macrophages was mediated by UCHL1 deletion.

### Deletion of UCHL1 induces downregulation of scavenger receptor CD36 protein

**Fig. 3:**
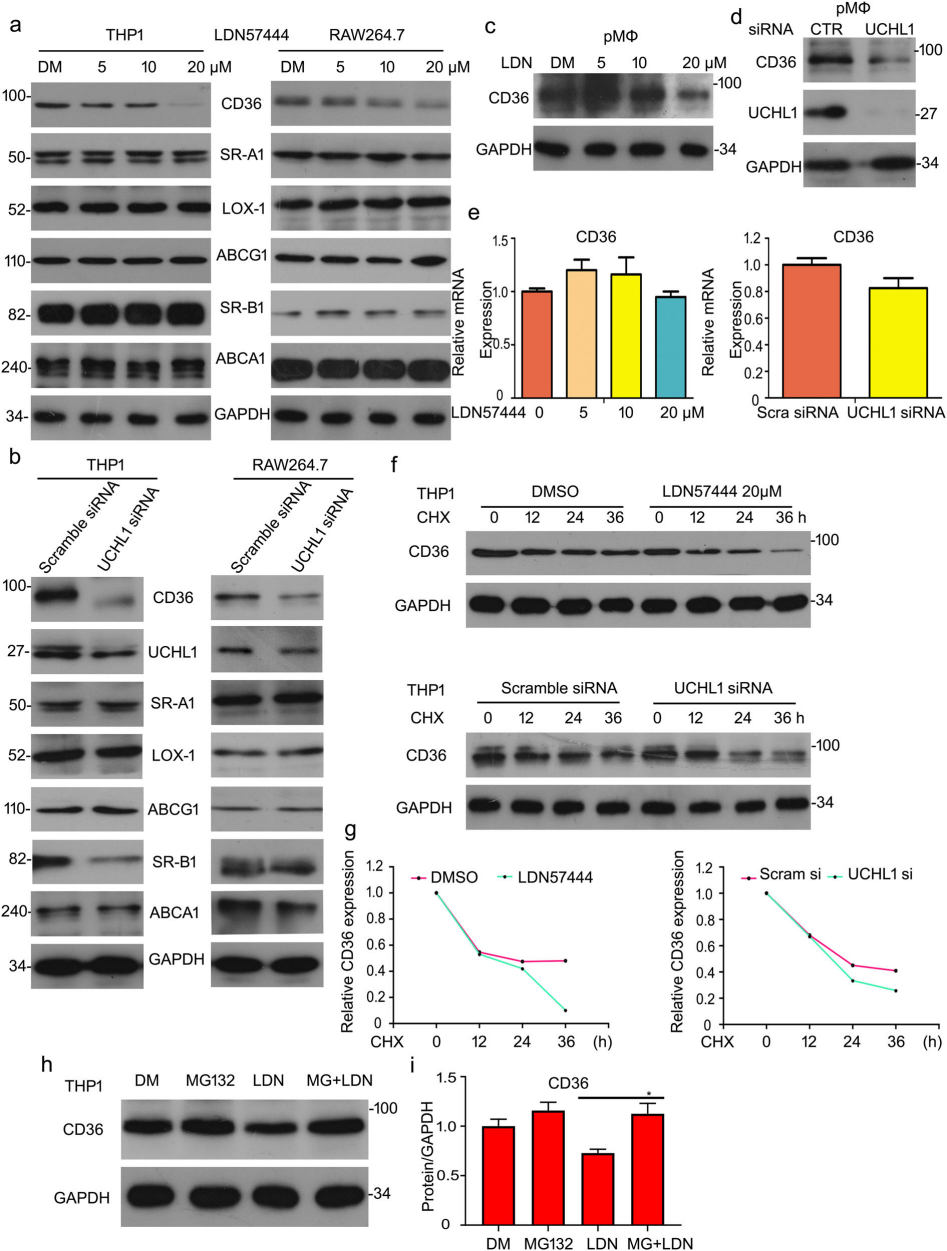
Deletion of UCHL1 induces downregulation of scavenger receptor CD36 protein. **a**, **b** THP1 and RAW264.7 cells were treated with LDN57444 (5, 10, 20 μM) or UCHL1 siRNA (50 nM) for the indicated time. Western blot analysis for SR-A, CD36, SR-B, ABCA1, ABCG1, and Lox-1. **c**, **d** Peritoneal macrophages (pMΦ) were treated with UCHL1 siRNA or LDN57444 and cells lysates were subjected to western blot assay for CD36. **e** The treated cells were subjected to RT-PCR analysis for CD36. **f** Macrophages were exposed to cycloheximide (CHX) for the indicated times and LDN57444 or UCHL1 siRNA in combination with CHX for the same time. Western blot analysis for CD36 expression. **h** THP1 cells were exposed to MG132 for 6 h and then added LDN57444 treatment for 24 h. The protein expression of CD36 was tested. GAPDH was as the loading control. **g**, **i** The bands of CD36 proteins were calculated. *p < 0.05 versus LDN57444 treatment group.

It is well known that oxLDL uptake and foam cell formation are dependent on ABC transporters and scavenger receptors, including ABCA1, ABCG1, Lox-1, SR-B1, SR-A, and CD36^7–12^. Given that UCHL1 induced the formation of foam cell, we supposed whether UCHL1 served as a mediator of scavenger receptors or ABC transporters. To verify the effect, we evaluated the expressions of CD36, SR-A, SR-B1, Lox-1, ABCA1, and ABCG1 proteins in THP1 and RAW264.7 cells. Western blot analysis indicated that UCHL1 inhibition or knockdown induced the downregulation of CD36 protein expression. While the other proteins have no significant change in THP1 and RAW264.7 cells (Fig. 3a, b). In addition, we harvested the mouse primary peritoneal macrophages to further assess the contribution of UCHL1 on the expression of CD36 protein. Significantly, the results showed that CD36 expression was decreased upon UCHL1 inhibition or silence (Fig. 3c, d). What is more, reverse transcription PCR (RT-PCR) results showed that UCHL1 deletion resulted in no meaning decreased in mRNA level of CD36 (Fig. 3e). We next explored the molecule mechanism of reduction of CD36 protein induced by UCHL1 inhibition or knockdown. The cycloheximide (CHX) was applied and the degradation of CD36 was measured by adding UCHL1 inhibitor or siRNA. The results of western blot assay revealed that inhibiting or silencing UCHL1 induced a decreased half-life of CD36 (Fig. 3f, g). To further explore the downregulation of CD36 and considering UCHL1 as a deubiquitinase, we supposed that UCHL1-promoted CD36 degradation was associated with proteasome system. MG132, which inhibits the 20S proteasome activity, was applied to asses CD36 expression. The results showed that the reduction of CD36 induced by inhibiting UCHL1 was rescued (Fig. 3h, i), indicating UCHL1 is essential for CD36 protein stability.

### Increased CD36 expression induced by oxLDL is regulated by UCHL1

**Fig. 4:**
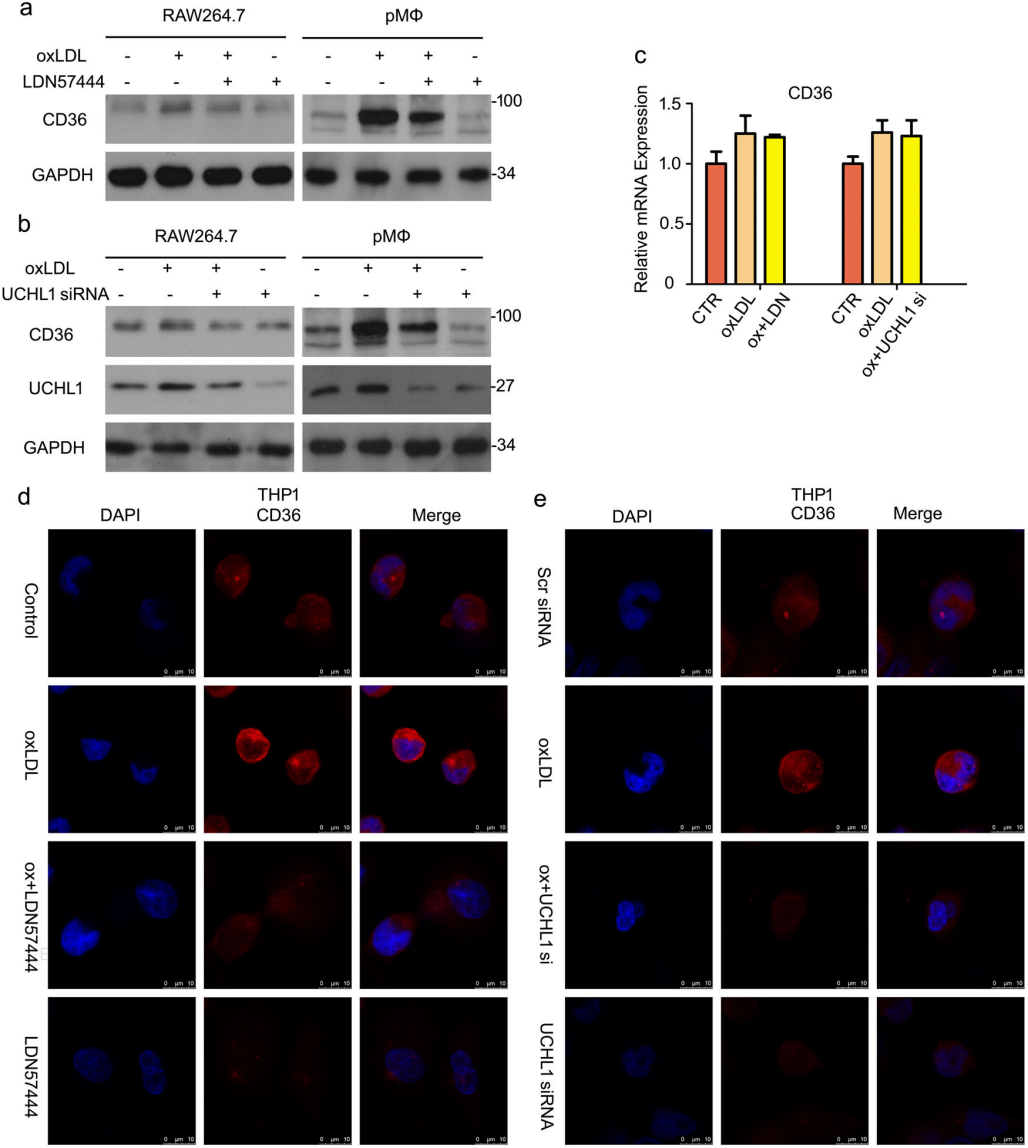
Increased CD36 expression induced by oxLDL is regulated by UCHL1. Macrophages were treated with oxLDL or/and LDN57444 for 24 h. Macrophages were treated with oxLDL or/and UCHL1 siRNA. **a**, **b** Cell lysates were collected and then subjected to western blot analysis for CD36. **c** The mRNA level of CD36 were detected using RT-qPCR. **d**, **e** Cell were fixed and incubated with CD36 antibody and secondary Cy3-conjugated antibody. The images were captured by confocal microscopy. The presented images were from at least three independent experiments.

It has been reported that oxLDL could activate CD36 expression^18^. Based on UCHL1-mediated lipid uptake via promoting CD36 stability, we assess whether UCHL1 inhibition induced downregulation of CD36 when oxLDL is existing. We found that oxLDL treatment significantly increased protein expression of CD36. Importantly, increased CD36 expression was inhibited by adding UCHL1 inhibitor or siRNA (Fig. 4a, b). RT-PCR analysis confirmed that oxLDL-promoted CD36 mRNA level did not regulated by UCHL1 inhibition (Fig. 4c). Moreover, we used immunofluorescent staining to observe CD36 expression and location. The results showed that CD36 expressed mainly in cytomembrane, suggesting CD36 is a membrane protein. Interestingly, we found that fluorescence intensity of CD36 was weakened in the group of UCHL1 inhibition in THP1 cells (Fig. 4d, e) and RAW264.7 and pMΦ (Supplementary Fig. S3). These results suggested that UCHL1 inhibition or knockdown suppressed protein expression of CD36 but not mRNA level, regardless of oxLDL stimulation.

### Molecular simulations for the interaction between CD36 and UCHL1

**Fig. 5:**
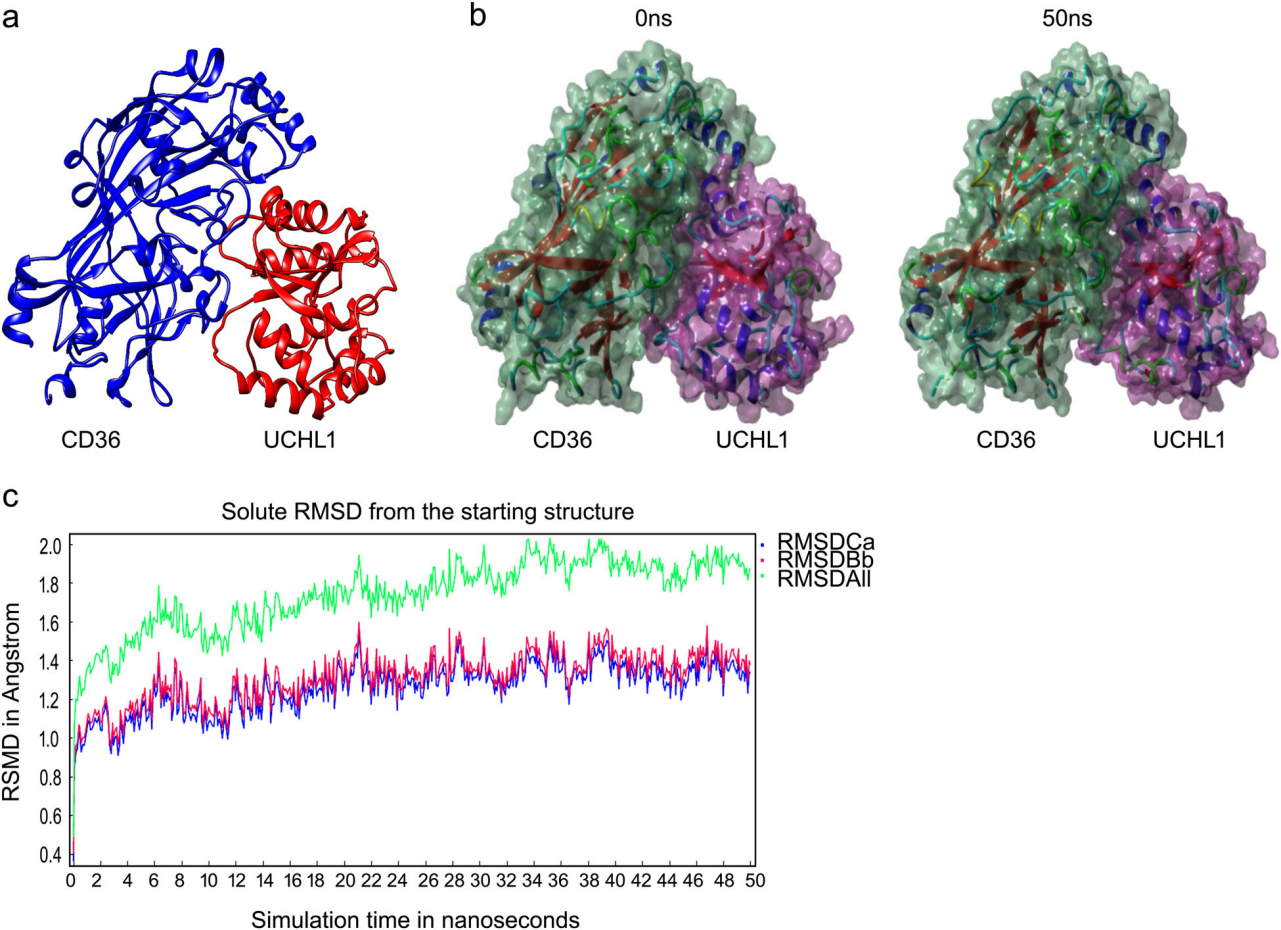
Molecular simulations for the interaction between CD36 and UCHL1. **a** Three-dimensional crystal structure of CD36–UCHL1 complex. **b** Surface presentation of the CD36–UCHL1 complex crystal structure at 0 and 50 ns. **c** Plots of root mean square deviation (RMSD) of Calpha atom (RMSDCa, blue), RMSD of backbone (RMSDBb, red), and RMSD of all-heavy atom (RMSDAll, green).

To further investigate the underlying mechanism of degradation of CD36 protein upon UCHL1 deletion, we assessed the interaction between UCHL1 and CD36. We used molecular simulations to observe the interaction of UCHL1 with CD36. As Fig. 5a shown, the three-dimensional binding conformation of UCHL1–CD36 complex was formed. To demonstrate the stability of this complex, we used molecular dynamics technique. The surface models of CD36–UCHL1 complex at 0 and 50 ns were observed. And CD36 interacts steadily UCHL1 until the end of MD simulation (Fig. 5b). In addition, the evaluation of atomic root mean square deviation (RMSD) of three proteins was shown, including Calpha atom RMSD (RMSDCa, blue), backbone atom RMSD (RMSDBb, red), all-heavy atom RMSD (RMSDAll, green). RMSDBb and RMSDCa track of CD36–UCHL1 complex rose from 0.1 to 1.2 Å during 6 ns, fluctuated around 1.2 Å until the end of MD simulation. RMSDAll track of CD36–UCHL1 complex rose from 0.1 to 1.8 Å during 6 ns, fluctuated around 1.8 Å until the end of MD simulation, suggesting the interaction of CD36–UCHL1 complex is steady (Fig. 5c).

### UCHL1 interacts with and stabilizes CD36 protein

**Fig. 6:**
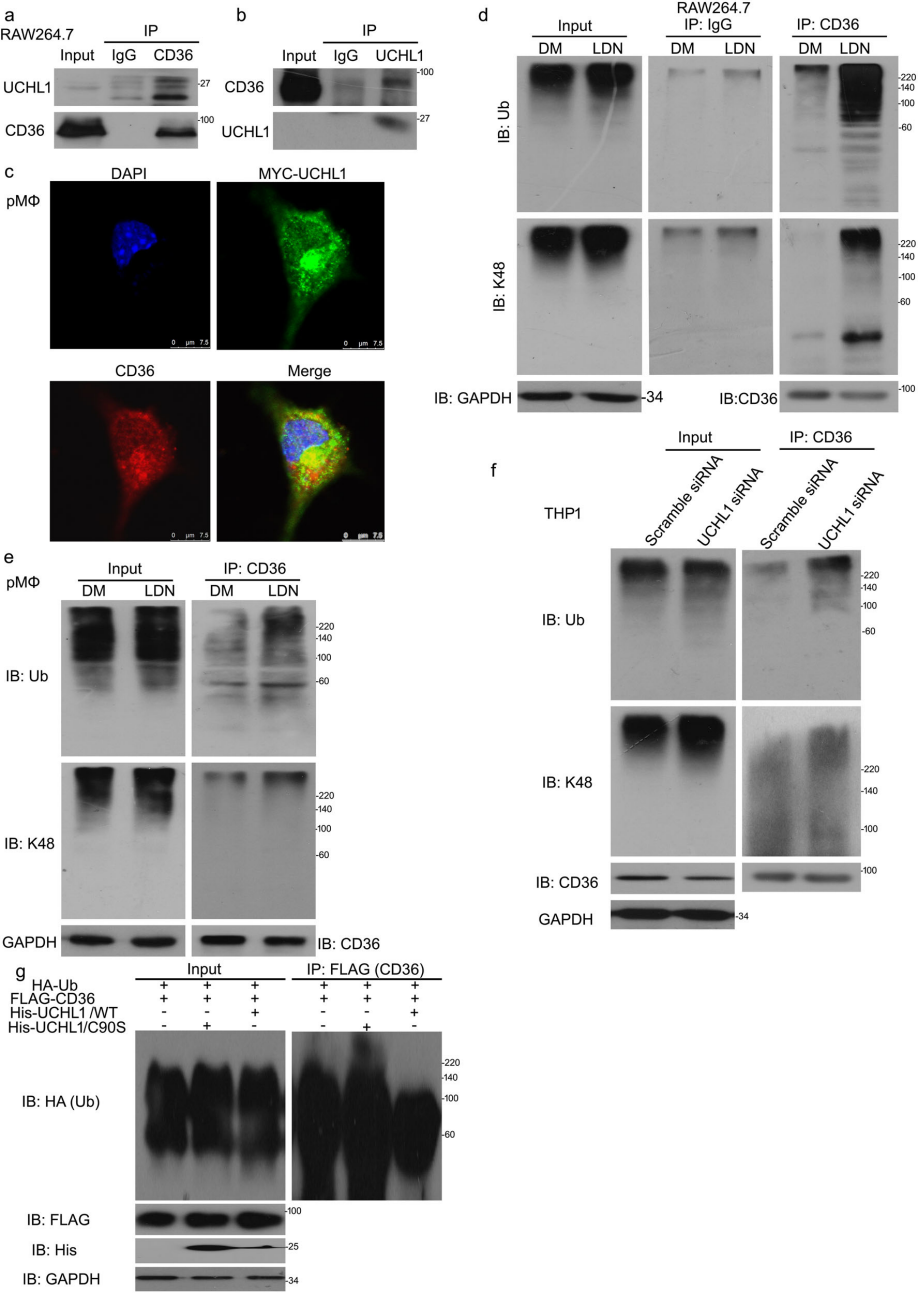
UCHL1 interacts with and stabilizes CD36 protein. **a** Immunoprecipitated with CD36 antibody beads for UCHL1, **b** immunoprecipitated with UCHL1 antibody beads for CD36 protein expression. **c** Peritoneal macrophages (pMΦ) were transfected with MYC-tagged UCHL1 for 48 h and incubated with MYC and CD36 antibodies. Confocal microscopy was used to test the location of MYC and CD36. **d**, **e** Macrophages were treated with LDN57444 for 24 h. Before harvested 6 h, MG132 were used to treated cells. **f** Macrophages were transfected with UCHL1 siRNA for 48 h and MG132 for 6 h. Western blot and Co-IP assays for polyubiquitin and K48-polyubiquitin expression. **g** HEK293T cells were transfected with FLAG-CD36, His-UCHL1/WT, His-UCHL1/C90S, and HA-Ub. Immunoprecipitated with FLAG and then immunoblotted with HA.

Coimmunoprecipitation (co-IP) with antibodies against CD36 followed by immunoblot (IB) with antibodies against UCHL1 showed that UCHL1 significantly interacted with CD36, and vice versa (Fig. 6a, b). In addition, we used immunofluorenscence staining to confirm direct interaction between these two proteins. pMΦ cells was transfected with MYC-tagged UCHL1 and subjected to stain with anti-MYC and anti-CD36 antibodies. Confocal assay showed that MYC-tagged UCHL1 appeared to be colocalized with endogenous CD36 in THP1 cells (Fig. 6c). We next evaluated whether the CD36–UCHL1 interaction has an impact on CD36 ubiquitination and the degradation of CD36 protein was a result of deubiquitination inhibition induced by UCHL1 deletion. We found that the level of poly-ubiquitinated CD36 was increased in UCHL1 inhibitor or siRNA treatment using co-IP assay (Fig. 6d–f). To further investigate that the decreased CD36 results from UCHL1 inhibition-promoted CD36 degradation, we performed the detection of expression of K48-poly-ubiquitinated CD36 (Fig. 6d–f). Western blot analysis showed that inhibiting or silencing UCHL1 markedly upregulated level of ubiquitinated CD36. More importantly, we generated HEK293T cells expressing His-tagged full-length UCHL1 (UCHL1/WT) and His-tagged catalytically inactive mutant of UCHL1 (UCHL1/C90S). Compared to UCHL1/WT, the abundance of poly-ubiquitinated CD36 was increased in cells expressing UCHL1/C90S (Fig. 6g), suggesting that the deubiquitinated enzymatic activity of UCHL1 is necessary for CD36 deubiquitination and stability.

### CD36 expression is indispensable for decreased lipid accumulation induced by deletion of UCHL1

**Fig. 7:**
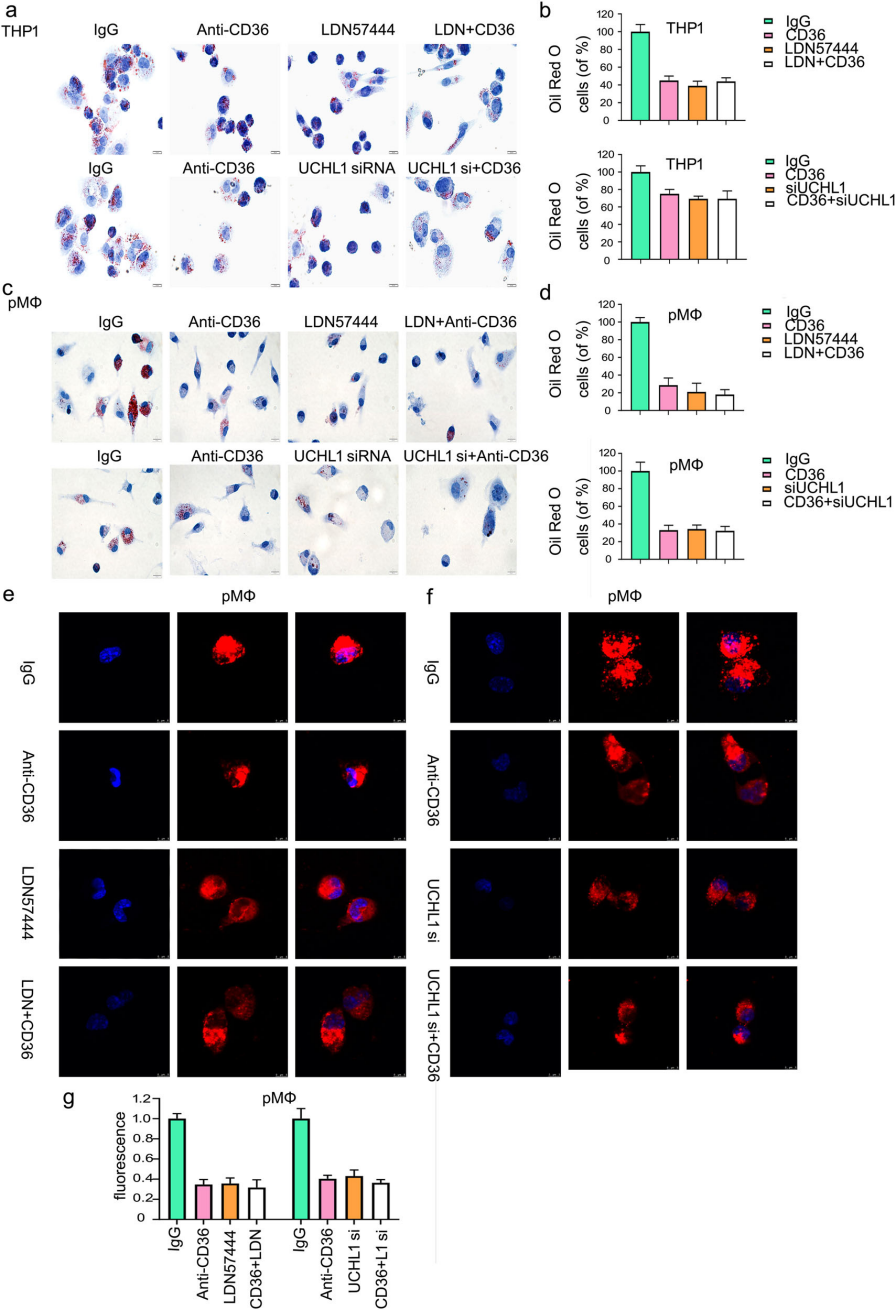
CD36 expression is indispensable for decreased lipid accumulation induced by deletion of UCHL1. Macrophages were pretreated with anti-CD36 or IgG antibody for 1 h, followed by exposed to either LDN57444 or UCHL1 siRNA for the indicated time. **a**, **c** OxLDL treatment for the last 24 h and macrophages were stained with oil red O. **e**, **f** Dil-oxLDL was used to treat cells for the additional 6 h, followed by confocal microscopy. The represented images were shown and from three independent experiments. **b**, **d** Quantitative analysis of Oil Red O-positive cells and **g** fluorescence intensity were present.

As we known, CD36 makes contribution to lipid uptake and foam cell formation. Blocking CD36 expression could inhibit 50% lipid uptake, which is a hallmark of atherosclerosis^13,14^. To further study the function of UCHL1 on the formation of foam cell, we applied the antibody-dependent blocking assay using anti-CD36 antibody incubation. Oil red O staining was applied to further determine the purpose that decreased lipid uptake is a consequence of the inhibition of UCHL1-induced downregulation of CD36. The results showed that UCHL1 deletion in combination with blocking CD36 expression has no significant change, compared with the signal treatment (Fig. 7a–d). In addition to oil red O staining, we found that lipid uptake was decreased after the incubation of anti-CD36 antibody in RAW264.7 and peritoneal macrophages using confocal microscopy. Moreover, UCHL1 inhibitor or siRNA-induced inhibition of lipid uptake has no marked difference by adding anti-CD36 antibody incubation (Fig. 7e–g; Supplementary Fig. S4).

## Discussion

Recruitment of immune cells to artery wall and lipid metabolism disfunction are the basic pathological progression of cerebrovascular and CAD disease. In the processes of atherogenesis, macrophages display membrane-bound lipid droplets through ingesting and processing lipoproteins in the cytoplasm. And then a foam cell phenotype is performed. Foam cell formation is related to the stability of atheromatous plaques and fatty streak formation. Foam cells have an important role in the processes of atherosclerosis. Increasing proatherogenic lipoproteins uptake and the downregulation of cleaning cholesterol from cells induced by a defective cholesterol efflux become the critical molecular mechanism of the formation of foam cell. Cholesterol efflux and lipid uptake are common causes of foam cell formation. Scavenger receptors, including SR-A, Lox-1, and CD36 promotes lipid uptake, and cholesterol efflux is regulated by scavenger receptor class B type 1 (SR-B1), and ATP-binding cassette transporters A1 and G1 (ABCA1 and ABCG1) having a negative role in foam cell formation.

It has been reported that scavenger receptors, CD36 and SR-A mediates roughly 75–90% of oxLDL uptake by macrophage and CD36 account for 50% of modified LDL binding in vitro^33^. CD36, one of scavenger receptor class B family, in addition to the major function in facilitating lipid uptake by macrophages, could bind some ligands, such as thrombospondin-1, oxLDL, apoptotic cells, and cell-derived microparticles^34^. The expression of macrophage CD36 is increased by inhibiting lysosomal and proteasome, suggesting the regulation of CD36 level associates with the ubiquitin pathway^20^.

Ubiquitination involves in the degradation of targeted proteins in eukaryotes by the function of the 26S proteasomes^35^. Ubiquitin is a protein of 76-residue and exist reversion to protein substrates by linkage between amino groups of lysine and the carboxyl group of ubiquitin on the acceptor proteins. Enzyme cascade facilitates ubiquitin molecules to link the substrate, which results in a rapid degradation of the substrate^36^. UCHL1, as a DUB, which promotes targeted protein stabilization, is reported that it is overexpressed in many organs and it has a major role in cancer treatment. We explored the possibility that ubiquitinated CD36 is regulated by UCHL1, which has an effect on the formation of foam cell.

Therefore, in current study, we firstly assessed the influence of UCHL1 on the formation of foam cell. Our findings is that UCHL1 inhibitor or knockdown suppresses atherogenic lipid oxLDL-induced foam cell formation in human THP1 macrophages, murine RAW264.7 macrophages and mouse primary peritoneal macrophages. Furthermore, UCHL1 have been regarded being related with lipid uptake. The newly indentified UCHL1 deficiency blocks lipid accumulation by macrophages, suggesting a potential link between UCHL1 and atherosclerosis. Mechanistically, this phenotype results from the decreased mRNA or protein levels of scavenger receptors or mediator of cholesterol transport. We firstly detected the proteins expression of CD36, SR-A, Lox-1, SR-B1, ABCG1, and ABCA1. Our study shows that inhibition of UCHL1 significantly decreases the CD36 expression but not regulate the other proteins with and without oxLDL stimulation. Next, we found that the mRNA level of CD36 is not affected by UCHL1, suggesting UCHL1-mediated CD36 expression is in the protein level but not in the mRNA level.

**Fig. 8:**
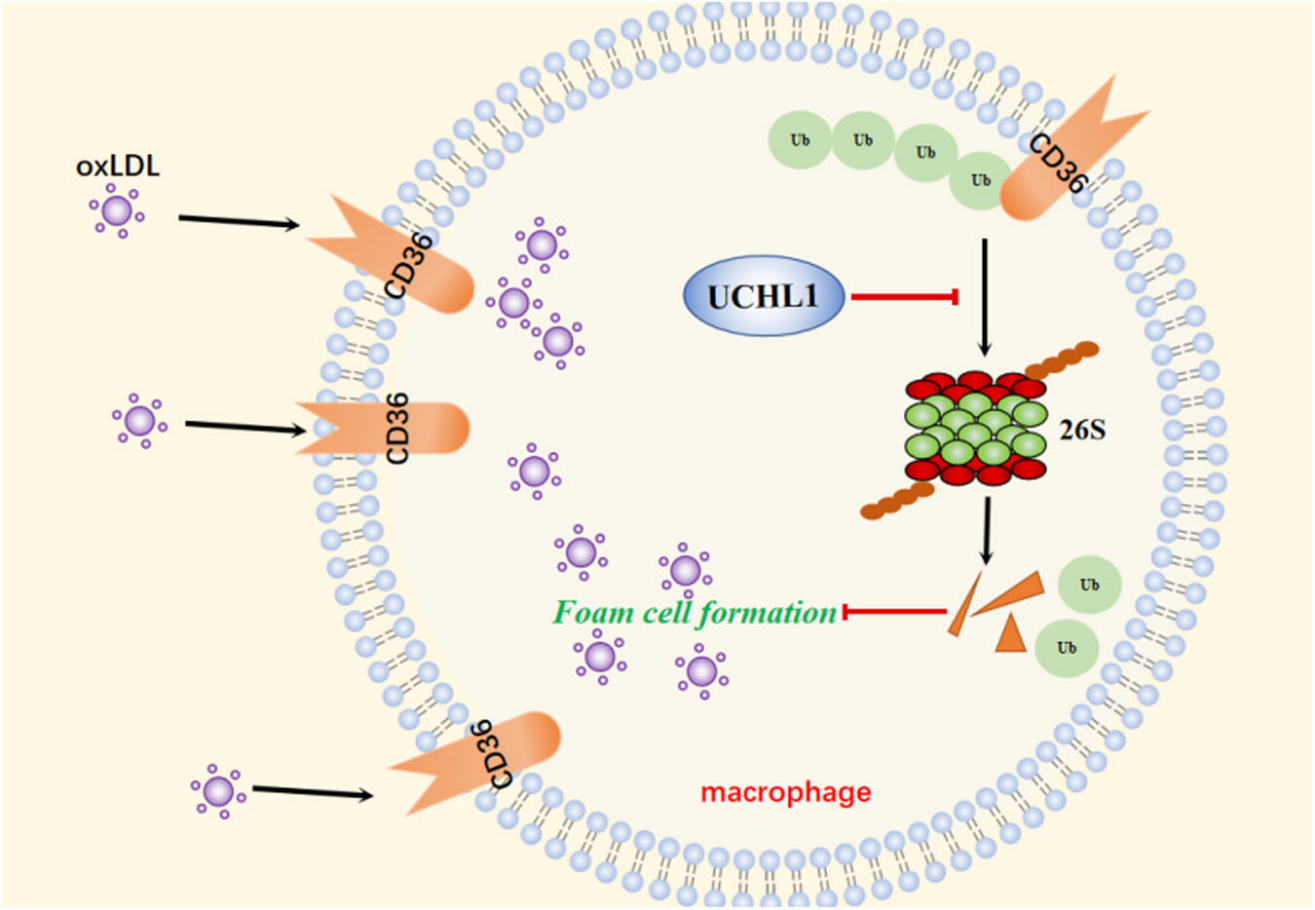
A proposed mechanism for UCHL1 to regulate foam cell formation via stabilizing the protein expression of CD36. OxLDL is uptaked by macrophages via scavenger receptor CD36 which locates in cell membrane, resulting in foam cell formation. UCHL1, as a deubiquitinating enzyme, blocks the ubiquitination progress of CD36 protein and then inhibiting its degradation. The stabilization of CD36 controlled by UCHL1 promoted foam cell formation in macrophages.

To further explore the molecular mechanism of UCHL1 on the regulation of CD36, we observed the inhibition of UCHL1 promotes CD36 degradation. Previous study has demonstrated that CD36 degradation involved in ubiquitin system^20^. We supposed whether decreased CD36 expression induced by UCHL1 is associated with UPS. MG132, a blocker in 20S, remarkably abrogates decreased CD36 expression upon UCHL1 deletion. Further findings confirmed that UCHL1 interacts with CD36 and its inhibition enhances the abundance of polyubiquitin and K48-polyubiquitin on CD36. Given that the function of UCHL1 in foam cell formation and CD36 regulation, we explored whether the inhibition of UCHL1-induced foam cell formation suppression is account for decreased CD36 expression. UCHL1 do not affect significantly lipid uptake and foam cell formation upon blocking CD36 expression. This results indicates that the suppression of lipid accumulation induced by UCHL1 inhibition or silence is a result of UCHL1 inhibition-promoted CD36 degradation (Fig. 8).

In conclusion, our study demonstrated that the inhibition of UCHL1 induces decreased oxLDL uptake and foam cell formation by macrophages via downregulating expression of CD36 protein, whereas it has no effect on cholesterol transport. Hence, this study provides a possible mechanism that UCHL1 mediates pathologic processes of atherosclerosis, indicating UCHL1 may be a therapeutic target in atherosclerosis treatment.

## Materials and methods

### Materials

LDN57444 (S7135) and MG132 (S2619) were from Selleckchem (Houston, TX, USA), dissolved into DMSO and keep at −20 °C. Control siRNA (SC-37007) and UCHL1 siRNA (SC-42304) was obtained from Santa Cruz Biotechnology (Santa Cruz, CA, USA). Human oxidized low-density lipoprotein (oxLDL) (YB-002) and Dil-labeled oxLDL (YB-0010) was from Yiyuan Biotechnologies (Guangzhou, China). Oil Red O was purchased from Sigma-Aldrich. All antibodies were purchased as followed: anti-CD36 (ab13365), anti-SR-B (ab21718), anti-SR-A (ab123946), Lox-1 (ab60178), and anti-ABCG1 (ab52617) were purchased Abcam. Anti-ubiquitin (3936), anti-K48 (12805), anti-GAPDH (5174), and anti-UCHL1 (13179) for western blot were from Cell Signaling Technology (MA, USA). Anti-Lox-1 was purchased from R&D system. Anti-ABCA1 (NB400105) and UCHL1 (NBP266760) for CO-IP were purchased from Novus Biologicals (USA).

### Cell lines and culture conditions

Human macrophage cell line THP1, murine macrophage cell line RAW264.7, and HEK293T were purchased from ATCC (Manassaa, VA, USA). DMEM (ThermoFisher Scientific), containing 10% FBS, was for RAW264.7 and HEK293T cells culture and RPMI 1640 medium, containing 10% FBS was for THP1 cells on 75 cm^2^ culture flask. THP1 cells were treated with PMA (200 ng/ml) for 24 h.

### Isolation of peritoneal macrophages and cell culture

Eight-week-old male C57BL mice were purchased from Guangdong Animal Center and sacrificed vertebral neck dislocation. Animal protocols were approved by the Institutional Animal Care and Use Committee of Guangzhou Medical University. Then 5 ml cold PBS was used to peritoneal injection and peritoneal fluids were collected from each mouse. Peritoneal macrophages (pMΦ) were centrifuged and harvested to plated into plates blindly, cultured with RPMI 1640 medium containing 10% FBS at 5% CO_2_ at 37 °C. After 4 h of pre-incubation, non-adherent cells were cleared and monolayer of pMΦ was identified by anti-F4/80 mAb with >95% staining.

### Foam cell formation assay

Macrophages cells were seeded into slides and exposed to LDN57444 for 24 h or UCHL1 siRNA for 48 h by incubating with oxLDL (50 μg/ml) for 24 h. Oil Red O, diluted to 0.5% in isopropyl alcohol, was prepared. Then cells were washed and fixed for 10 min, followed by the mixture of water: 0.5% Oil Red O (2:3) for 3–5 min at room temperature. Cells were washed with 60% isopropanol once and water for two times. Hematoxylin was used to stain cell for 3 s and running water for washing. Images were taken by optical microscope.

### Dil-oxLDL uptake assay

Macrophage cells were collected and seeded into confocal cuvette for 24 h. Cells were treated with UCHL1 inhibitor or siRNA for the indicated time. Fluorescence-labeled oxidative LDL was used for additional 6 h in 37 °C. For functional neutralizing assay, cells were pretreated with anti-IgG or anti-CD36 antibody for 1 h. And then cells were exposed to inhibitor or siRNA and Dil-oxLDL. Macrophage cells were washed with PBS and fixed with 4% paraformaldehyde. Cell nucleus were stained with DAPI (blue). Images were captured using fluorescence microscope and stained cells were calculated by Image Pro Plus software.

### Western blot and co-IP assay

Protein levels were determined as described before^37^. Cells were treated with the indicated treatment. Total lysates were harvested, followed by SDS-PAGE. For protein interaction (co-IP) analysis, reported in our previous study^38,39^, antibodies and dynabeads were mixtured for 16–24 h. Cell lysates from cells were used to react with the mixture of antibodies and dynabeads, and rotated for 1 h. The mixtures were washed with PBS-T and then loading buffer was used to suspended, followed by western blot.

### Plasmids and transfection of cells

The assay was performed as we previously reported^40,41^. The plasmid His/MYC-UCHL1, His-UCHL1 (C90S), HA-Ub, and FLAG-CD36 encoding fusion proteins were purchased from Genechem (Shanghai, China). pMΦ or HEK293T cells were seeded into plates. Then cells were culture with the mixture of plasmid or control vector and lipofectamine 3000 reagent (Life Technologies) for 48 h.

### Flow cytometry assay

Cells were treated with UCHL1 inhibitor or siRNA and incubated for Dil-oxLDL for the additional 6 h. After then, cells were washed with PBS for three times and digested with pancreatic enzymes. Collected cells were suspended with PBS, subjected to flow cytometry analysis.

### Immunofluorescence assay

The intensity of fluorescence were evaluated as we reported before^42,43^. Cells were digested and plated onto chamber slide. Then cells were exposed to the UCHL1 inhibitor or siRNA for the indicated time. 0.1% Triton X-100 diluted in PBS was used for 10 min and then 5% BSA were used for 30 min to incubate cells. Cells were cultured with primary antibody for 1 h and then secondary Cy3-conjugated antibody for 1 h. PBS was used to wash cells for three times each time. Lastly, cells were added with fluoroshied mounting medium with DAPI (Abcam). Images were captured using confocal microscope.

### PCR assay

The assay was performed as before reported in our study^44^. Cells were treated and then washed using PBS. Trizol reagent (Invitrogen) was used to perform total RNA harvest. The concentration and purity of RNA were determined at 260:280 nm. According to the manufacturer’s instructions of PrimeScript II 1st Strand cDNA Synthesis Kit (TaKaRa), the first-strand cDNA were synthesized upon an equal amount of RNAs. Real-time quantitative PCR was used to test mRNA levels. PCR primes are as following: CD36: forward: 5′-TTTCCTCTGACATTTGCAGGTCTA-3′ and reverse: 5′-AAAGGCATTGGCTGGAAGA-3′; GAPDH: forward:5′-ACCCAGAAGACTGTGGATGG-3′ and reverse: 5′-ACACATTGGGGGTAGGAACA-3′.

### SiRNA transfection

The transfection was performed as we previously described^45^. THP1 cells were seeded in dishes and treated with PMA for 24 h. The mixture, containing RPMI opti-MEM, siRNs, and lipofectamine RNAiMax (Invitrogen) reagent, were prepared and added in dishes and cultured for 48 h.

### Molecular dynamics simulation

Docking analysis was performed by PatchDock (https://bioinfo3d.cs.tau.ac.il/PatchDock/). The crystal structure of CD36 (PDB ID: 5LGD) and UCHL1 (PDB ID: 4DM9) were obtained from protein data bank (http://www.rcsb.org/). The ligand molecular selection tab was applied for the upload of pdb files in PatchDock. The geometric shape complementarity score was used to asses binding mode and binding affinity. The best conformations were applied as the starting conformation for MD simulation. YASARA was taken for molecular dynamics simulation. All simulations were performed by AMBER 03 forcefield. Specifically, solvation of the protein complex using 0.9% NaCl carrying a distance of 5 Å between the solute and box in a dodecahedron box. 298K was regarded as the initiation of simulated annealing minimizations, through velocities scaling down by 0.9 every ten steps lasting for 5 ps. When energy was becoming minimized, Berendsen thermostat was applied to decrease the temperature-induced influence, temperature of the system was modulated. What is the more, velocities were modulated only every 100 simulation steps, with temperatures converged from the mean of the last 100 detected. Lastly, a rate of 2 fs was applied for 100 ns MD simulations, and the coordinates of the complexes were saved every 10 ps.

### Statistical assay

The showed data are as mean ± SD. The unpaired Student’s *t*-test were applied to analysis the statistical significance of differences. All statistical analyses were performed using SPSS 22.0 and GraphPad Prism 5. The level of *p* < 0.05 was accepted.

## Supplementary information

Supplementary Figure Legends

Figure S1

Figure S2

Figure S3

Figure S4

## References

[1] Vasan RS, Benjamin EJ (2016). The future of cardiovascular epidemiology. Circulation.

[2] Writing Group M (2016). Heart disease and stroke statistics-2016 update: a report from the American Heart Association. Circulation.

[3] Go AS (2013). Executive summary: heart disease and stroke statistics-2013 update: a report from the American Heart Association. Circulation.

[4] Legein B, Temmerman L, Biessen EA, Lutgens E (2013). Inflammation and immune system interactions in atherosclerosis. Cell. Mol. Life Sci..

[5] Yuan Y, Li P, Ye J (2012). Lipid homeostasis and the formation of macrophage-derived foam cells in atherosclerosis. Protein Cell.

[6] Moore KJ, Tabas I (2011). Macrophages in the pathogenesis of atherosclerosis. Cell.

[7] Chistiakov DA, Bobryshev YV, Orekhov AN (2016). Macrophage-mediated cholesterol handling in atherosclerosis. J. Cell Mol. Med..

[8] Kunjathoor VV (2002). Scavenger receptors class A-I/II and CD36 are the principal receptors responsible for the uptake of modified low density lipoprotein leading to lipid loading in macrophages. J. Biol. Chem..

[9] Li AC, Glass CK (2002). The macrophage foam cell as a target for therapeutic intervention. Nat. Med..

[10] Sporstol M, Mousavi SA, Eskild W, Roos N, Berg T (2007). ABCA1, ABCG1 and SR-BI: hormonal regulation in primary rat hepatocytes and human cell lines. BMC Mol. Biol..

[11] Tall AR, Yvan-Charvet L, Terasaka N, Pagler T, Wang N (2008). HDL, ABC transporters, and cholesterol efflux: implications for the treatment of atherosclerosis. Cell Metab..

[12] Yvan-Charvet L, Wang N, Tall AR (2010). Role of HDL, ABCA1, and ABCG1 transporters in cholesterol efflux and immune responses. Arterioscler. Thromb. Vasc. Biol..

[13] Endemann G (1993). CD36 is a receptor for oxidized low density lipoprotein. J. Biol. Chem..

[14] Rahaman SO (2006). A CD36-dependent signaling cascade is necessary for macrophage foam cell formation. Cell Metab..

[15] Nicholson AC, Frieda S, Pearce A, Silverstein RL (1995). Oxidized LDL binds to CD36 on human monocyte-derived macrophages and transfected cell lines. Evidence implicating the lipid moiety of the lipoprotein as the binding site. Arterioscler. Thromb. Vasc. Biol..

[16] Nozaki S (1995). Reduced uptake of oxidized low density lipoproteins in monocyte-derived macrophages from CD36-deficient subjects. J. Clin. Invest..

[17] Febbraio M (2000). Targeted disruption of the class B scavenger receptor CD36 protects against atherosclerotic lesion development in mice. J. Clin. Invest..

[18] Han J, Hajjar DP, Febbraio M, Nicholson AC (1997). Native and modified low density lipoproteins increase the functional expression of the macrophage class B scavenger receptor, CD36. J. Biol. Chem..

[19] Smith J, Su X, El-Maghrabi R, Stahl PD, Abumrad NA (2008). Opposite regulation of CD36 ubiquitination by fatty acids and insulin: effects on fatty acid uptake. J. Biol. Chem..

[20] Sun S (2018). Ubiquitinated CD36 sustains insulin-stimulated Akt activation by stabilizing insulin receptor substrate 1 in myotubes. J. Biol. Chem..

[21] Zhang F (2020). Inhibition of USP14 suppresses the formation of foam cell by promoting CD36 degradation. J. Cell Mol. Med..

[22] D’Arcy P, Linder S (2014). Molecular pathways: translational potential of deubiquitinases as drug targets. Clin. Cancer Res..

[23] Tian Z (2014). A novel small molecule inhibitor of deubiquitylating enzyme USP14 and UCHL5 induces apoptosis in multiple myeloma and overcomes bortezomib resistance. Blood.

[24] Larsen CN, Price JS, Wilkinson KD (1996). Substrate binding and catalysis by ubiquitin C-terminal hydrolases: identification of two active site residues. Biochemistry.

[25] Liu Y, Fallon L, Lashuel HA, Liu Z, Lansbury PT (2002). The UCH-L1 gene encodes two opposing enzymatic activities that affect alpha-synuclein degradation and Parkinson’s disease susceptibility. Cell.

[26] Setsuie R, Wada K (2007). The functions of UCH-L1 and its relation to neurodegenerative diseases. Neurochem. Int..

[27] Goto Y (2015). UCHL1 provides diagnostic and antimetastatic strategies due to its deubiquitinating effect on HIF-1alpha. Nat. Commun..

[28] Hussain S (2010). The de-ubiquitinase UCH-L1 is an oncogene that drives the development of lymphoma in vivo by deregulating PHLPP1 and Akt signaling. Leukemia.

[29] Jin C (2013). UCHL1 is a putative tumor suppressor in ovarian cancer cells and contributes to cisplatin resistance. J. Cancer.

[30] Li L (2010). The tumor suppressor UCHL1 forms a complex with p53/MDM2/ARF to promote p53 signaling and is frequently silenced in nasopharyngeal carcinoma. Clin. Cancer Res..

[31] Ummanni R (2011). Ubiquitin carboxyl-terminal hydrolase 1 (UCHL1) is a potential tumour suppressor in prostate cancer and is frequently silenced by promoter methylation. Mol. Cancer.

[32] Zhong J (2012). UCHL1 acts as a colorectal cancer oncogene via activation of the beta-catenin/TCF pathway through its deubiquitinating activity. Int. J. Mol. Med..

[33] Nicholson AC, Han J, Febbraio M, Silversterin RL, Hajjar DP (2001). Role of CD36, the macrophage class B scavenger receptor, in atherosclerosis. Ann. N. Y. Acad. Sci..

[34] Febbraio M, Silverstein RL (2007). CD36: implications in cardiovascular disease. Int. J. Biochem. Cell Biol..

[35] Hochstrasser M (1996). Ubiquitin-dependent protein degradation. Annu. Rev. Genet..

[36] Miranda M, Sorkin A (2007). Regulation of receptors and transporters by ubiquitination: new insights into surprisingly similar mechanisms. Mol. Interv..

[37] Hu M (2018). The harsh microenvironment in infarcted heart accelerates transplanted bone marrow mesenchymal stem cells injury: the role of injured cardiomyocytes-derived exosomes. Cell Death Dis..

[38] Liao Y (2020). Targeting GRP78-dependent AR-V7 protein degradation overcomes castration-resistance in prostate cancer therapy. Theranostics.

[39] Xia X (2019). Deubiquitination and stabilization of estrogen receptor alpha by ubiquitin-specific protease 7 promotes breast tumorigenesis. Cancer Lett..

[40] Liao Y (2017). Proteasome-associated deubiquitinase ubiquitin-specific protease 14 regulates prostate cancer proliferation by deubiquitinating and stabilizing androgen receptor. Cell Death Dis..

[41] Liao Y (2018). Growth arrest and apoptosis induction in androgen receptor-positive human breast cancer cells by inhibition of USP14-mediated androgen receptor deubiquitination. Oncogene.

[42] Liu N (2019). Auranofin lethality to prostate cancer includes inhibition of proteasomal deubiquitinases and disrupted androgen receptor signaling. Eur. J. Pharmacol..

[43] Xia X (2018). Targeting proteasome-associated deubiquitinases as a novel strategy for the treatment of estrogen receptor-positive breast cancer. Oncogenesis.

[44] Liao Y (2019). USP10 modulates the SKP2/Bcr-Abl axis via stabilizing SKP2 in chronic myeloid leukemia. Cell Discov..

[45] Xu R (2019). Exosomes derived from pro-inflammatory bone marrow-derived mesenchymal stem cells reduce inflammation and myocardial injury via mediating macrophage polarization. J. Cell Mol. Med..

