# Supplementary material for: Deubiquitination of CD36 by UCHL1 promotes foam cell formation
Source: Cell Death Dis. 2020 Aug 15;11(8):636. doi: 10.1038/s41419-020-02888-x (PMC7429868; doi:10.1038/s41419-020-02888-x)
Supplement: Supplementary file 1 — Supplementary Figure Legends [file 41419_2020_2888_MOESM1_ESM.docx]

**Fig. S1. Mouse primary peritoneal macrophages (pMΦ) were identified.** Macrophages were incubated with anti-mouse F4/80 diluted in BSA (1:100) for 30 min, followed by fluorescence microscope.

**Fig. S2. UCHL1 deletion induces the inhibition of lipid uptake.** RAW264.7 cells were treated with LDN57444 (**a**) or UCHL1 siRNA (**b**) for the indicated time and then incubated with Dil-oxLDL, followed by confocal microscopy. (**c**) Quantitative analysis of fluorescence intensity were present. **p<0.01 *versus* oxLDL treatment group.

**Fig. S3. UCHL1 inhibition suppresses the expression of CD36.** Macrophages exposed to oxLDL, were treated with LDN57444 (**a**) or UCHL1 siRNA (**b**), followed by confocal microscopy.

**Fig. S4. CD36 expression is indispensable for decreased lipid accumulation induced by deletion of UCHL1.** Macrophages were pretreated with anti-CD36 or IgG antibody for 1 h, followed by exposed to either LDN57444 or UCHL1 siRNA for the indicated time. (**a, b**) Dil-oxLDL was used to treat cells for the additional 6 h, followed by confocal microscopy. (**c**) Quantitative analysis of fluorescence intensity were present.
