# Supplementary figures and images for: Deubiquitination of CD36 by UCHL1 promotes foam cell formation
Source: Cell Death Dis. 2020 Aug 15;11(8):636. doi: 10.1038/s41419-020-02888-x (PMC7429868; doi:10.1038/s41419-020-02888-x)

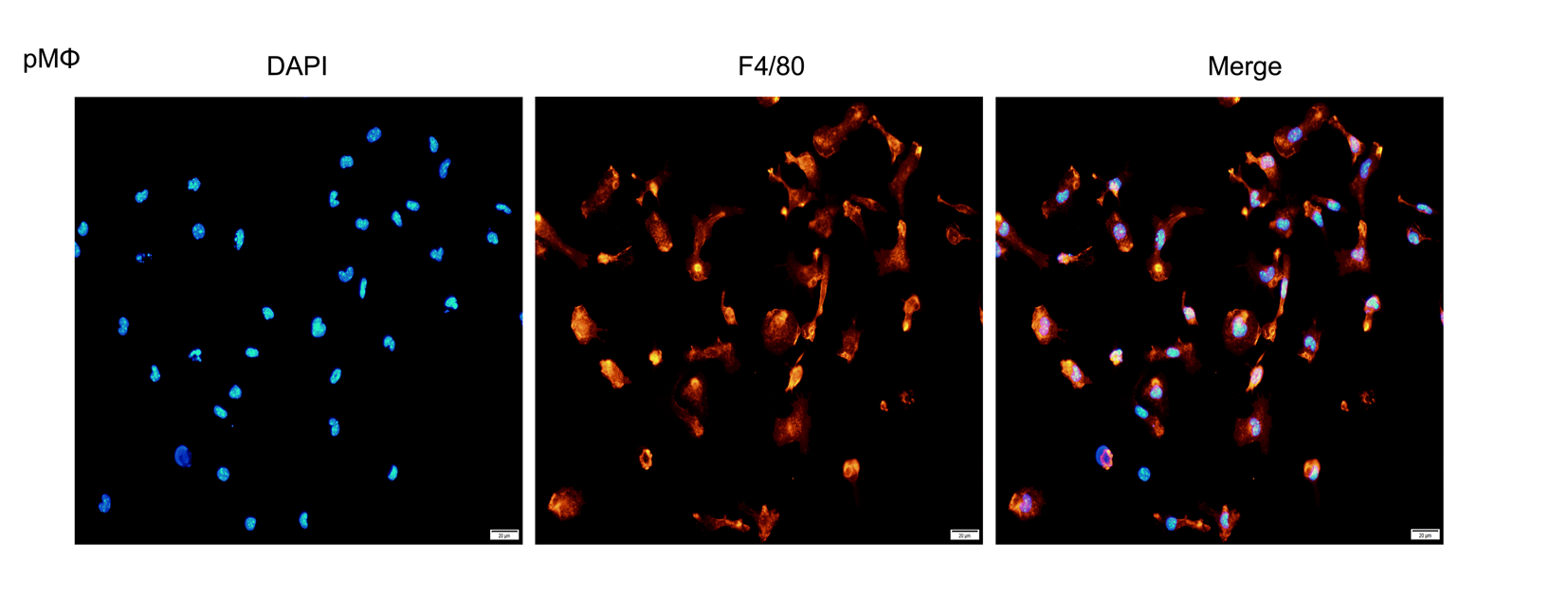

Supplement: Supplementary file 2 — Figure S1 [file 41419_2020_2888_MOESM2_ESM.tif]

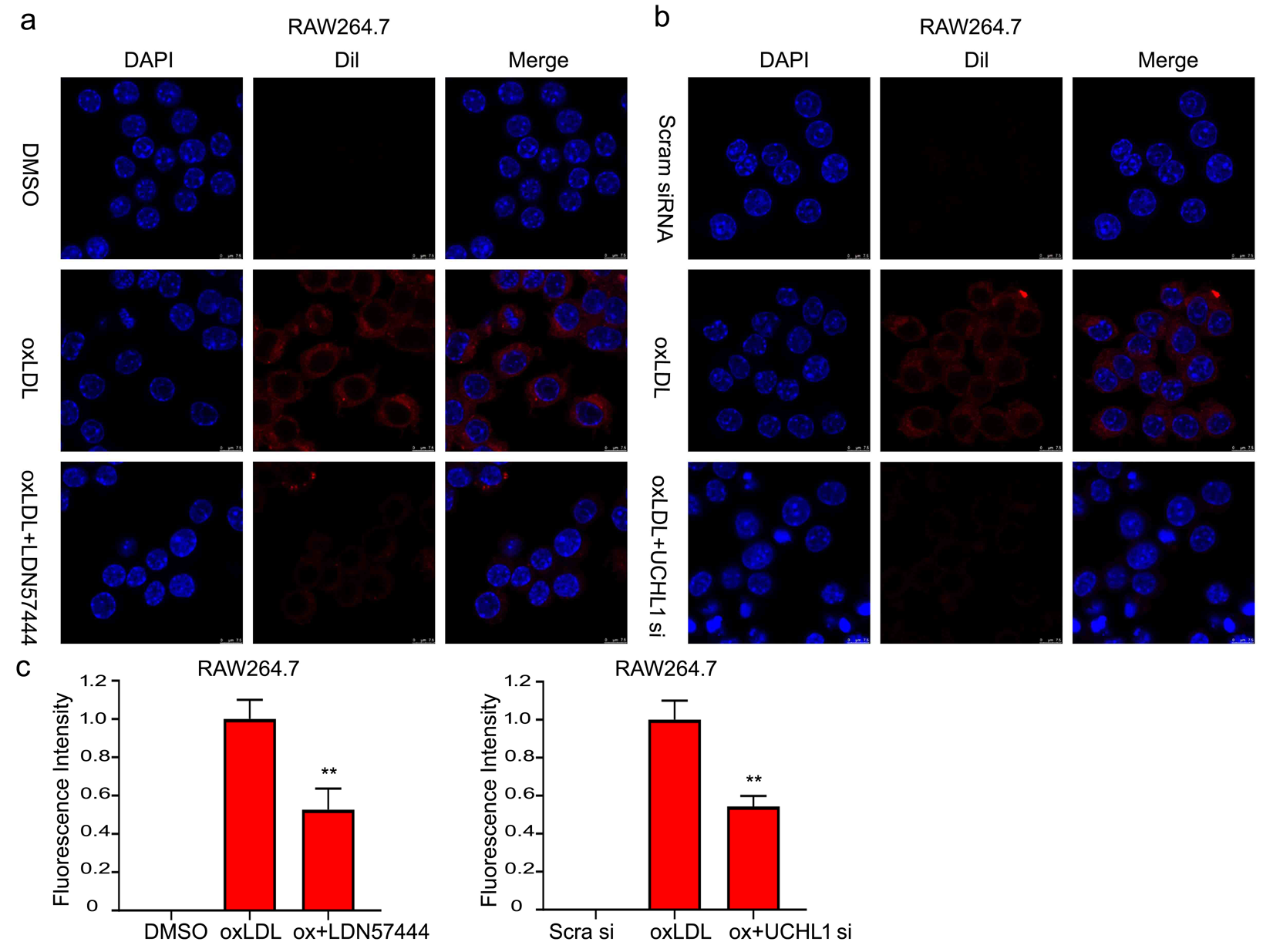

Supplement: Supplementary file 3 — Figure S2 [file 41419_2020_2888_MOESM3_ESM.tif]

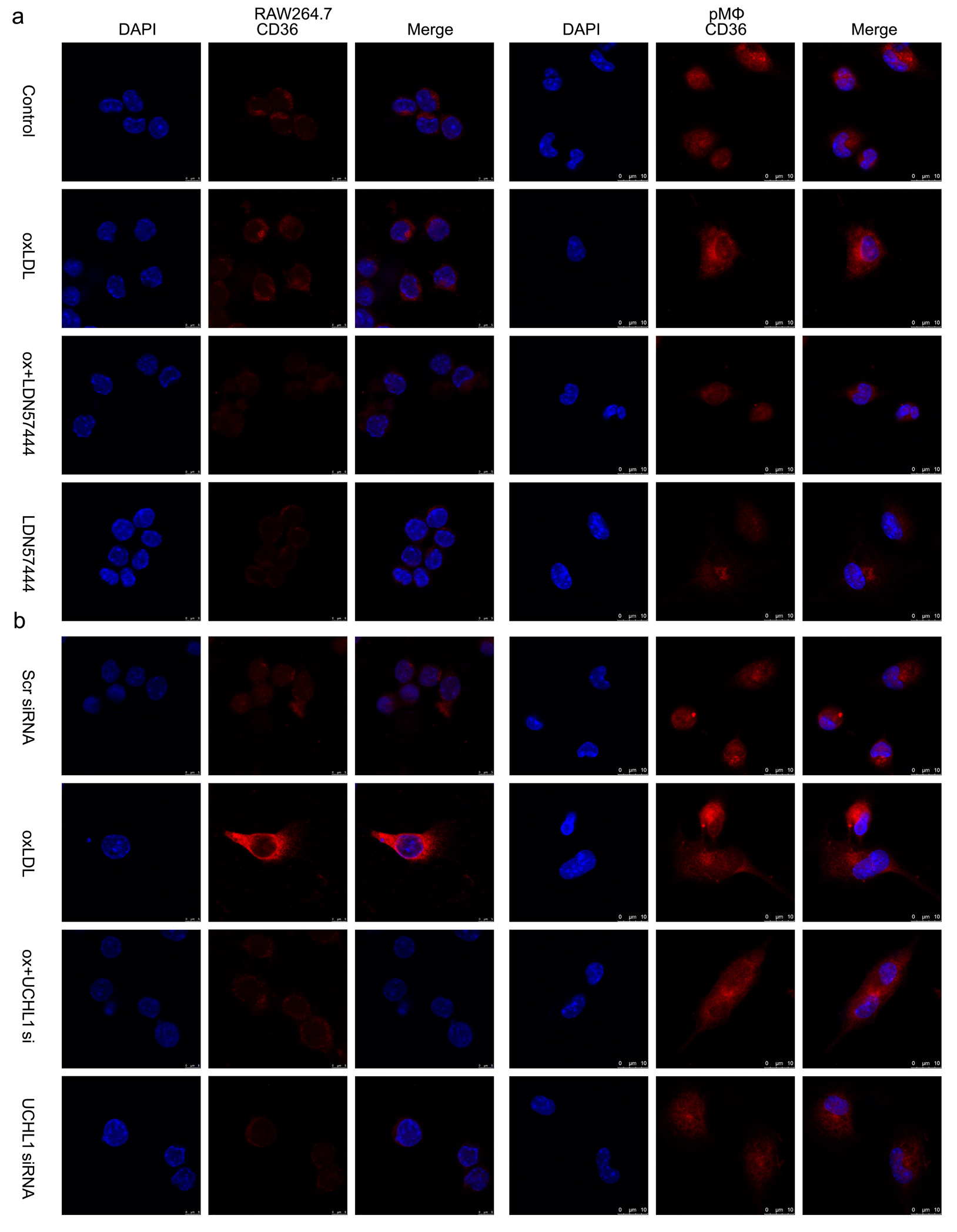

Supplement: Supplementary file 4 — Figure S3 [file 41419_2020_2888_MOESM4_ESM.tif]

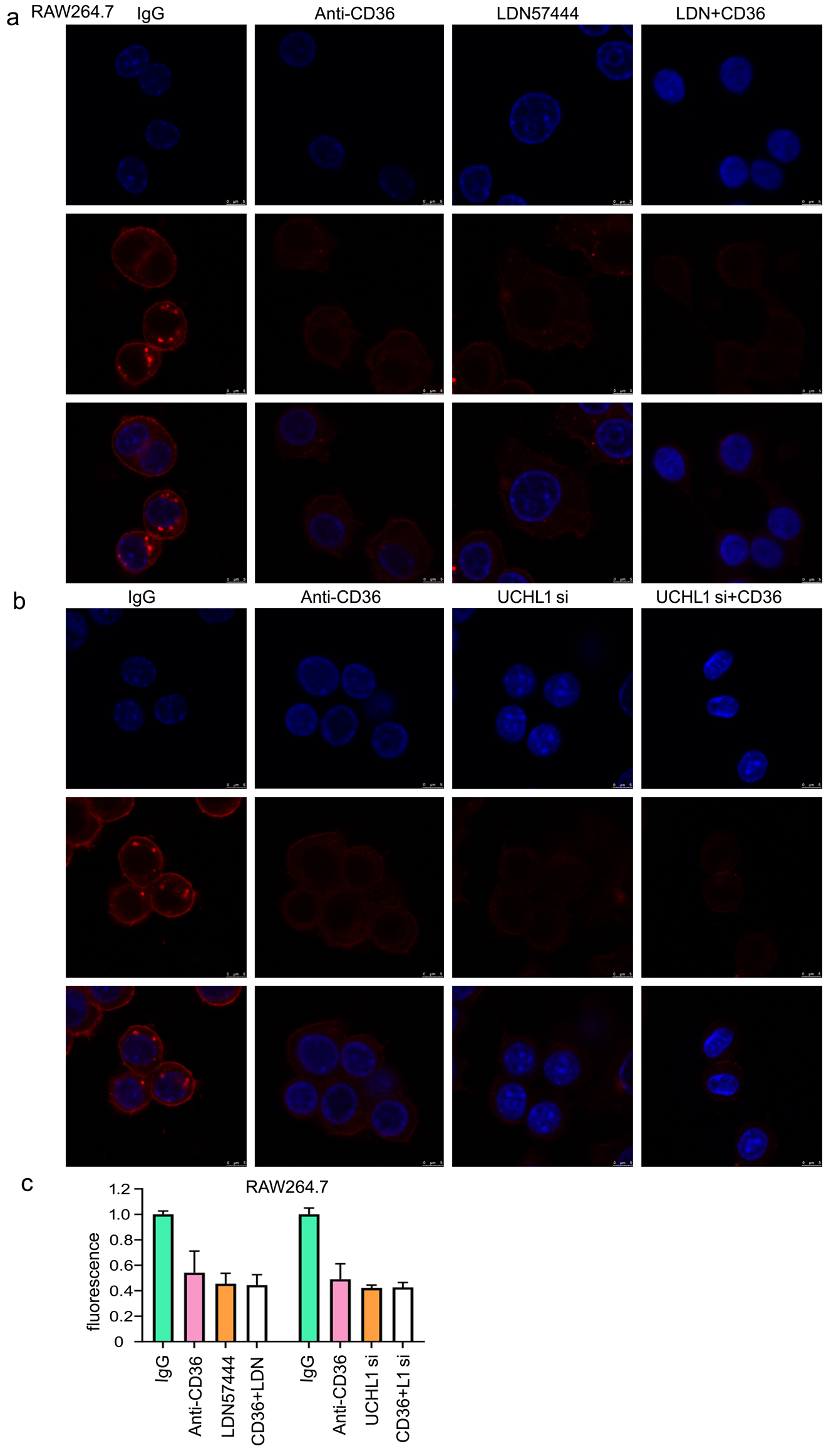

Supplement: Supplementary file 5 — Figure S4 [file 41419_2020_2888_MOESM5_ESM.tif]
